# Awareness and use of home remedies in Italy’s alps: a population-based cross-sectional telephone survey

**DOI:** 10.1186/s12906-022-03781-0

**Published:** 2022-11-11

**Authors:** Wolfgang Wiedermann, Dietmar Ausserhofer, Anna Vögele, Ulrich Becker, Giuliano Piccoliori, Christian J. Wiedermann, Adolf Engl

**Affiliations:** 1 Department of Educational, School and Counseling Psychology, College of Education and Human Development, Missouri Prevention Science Institute, University of Missouri, Columbia, MO USA; 2 Institute of General Practice and Public Health, Claudiana - College of Health Professions, Bolzano, Italy; 3 Institute of Nursing Science, Department Public Health, University of Basel, Basel, Switzerland; 4 Apollis Institute of Social Research and Opinion Polling, Bolzano, Italy; 5 Department of Public Health, Medical Decision Making and HTA, University of Health Sciences, Medical Informatics and Technology – Tyrol, Hall, Tyrol, Austria

**Keywords:** Language group, Vaccine hesitancy, Complementary and alternative medicine, Latent class analysis

## Abstract

**Background:**

Belief in complementary and alternative medicine practices is related to reduced preparedness for vaccination. This study aimed to assess home remedy awareness and use in South Tyrol, where vaccination rates in the coronavirus pandemic were lowest in Italy and differed between German- and Italian-speaking inhabitants.

**Methods:**

A population-based survey was conducted in 2014 and analyzed using descriptive statistics, multiple logistic regression, and latent class analysis.

**Results:**

Of the representative sample of 504 survey respondents, 357 (70.8%) participants (43.0% male; primary language German, 76.5%) reported to use home remedies. Most commonly reported home remedies were teas (48.2%), plants (21.0%), and compresses (19.5%). Participants from rural regions were less likely (odds ratio 0.35, 95% confidence interval 0.19–0.67), while female (2.62, 1.69–4.10) and German-speaking participants (5.52, 2.91–9.88) were more likely to use home remedies. Latent classes of home remedies were “alcoholic home remedies” (21.4%) and “non-alcohol-containing home remedies” (78.6%). Compared to the “non-alcohol-containing home remedies” class, members of the “alcoholic home remedies” class were more likely to live in an urban region, to be male and German speakers.

**Conclusion:**

In addition to residence and sex, language group membership associates with awareness and use of home remedies. Home remedies likely contribute to socio-cultural differences between the language groups in the Italian Alps. If the observed associations explain the lower vaccination rates in South Tyrol among German speakers requires further study.

**Supplementary Information:**

The online version contains supplementary material available at 10.1186/s12906-022-03781-0.

## Introduction

On December 16, 2021, The New York Times reported that “in Italyʼs alps, traditional medicine flourishes, as does Covid-19”, because the province of Bolzano had the countryʼs highest level of coronavirus infection and lowest vaccination rate, which the article related to the suggestion that “many people there prefer to rely on the pure air and herbal remedies” [1]. South Tyrol, the Autonomous Province of Bolzano, is a northwestern region of Italy in the southern alps next to Austria (total population, 524.256) with approximately 70% German and 25% Italian-speaking inhabitants [2]. The different language groups are served by the same healthcare system and service provider. Of all Italian regions, South Tyrol was in the worst position in December 2021 in terms of both vaccination rates and infection rates in the pandemic, despite the fact that the health system in South Tyrol is considered better than in many other regions [3]. The majority of the South Tyrolean population has German as its mother tongue and it was found that the Italian language group was more likely to follow prescribed pandemic control measures. In the group of teachers, for example, only 3 per cent of Italian speakers were not vaccinated, compared to 20 per cent of German speakers [4]. It was suspected that the German-speaking South Tyroleans might have less confidence in the stately institutions and recommendations, because in the historical development the demarcation from Austria could still cause a sceptical attitude towards the Italian central government. In fact, a May 2021 pandemic survey showed that 70 per cent of Italian speakers trusted the recommendations of the Italian National Institute of Health, but only 44 per cent of German speakers [4]. South Tyrol had low levels of childhood vaccination before coronavirus pandemic, with instead of the range of 90% only 71.9% of children 24 months of age jabbed against measles in 2017 [5].

Herbal medicine and other home remedies play an important role in public beliefs about effective prevention and treatment of diseases including the coronavirus disease 2019 (COVID-19) [6]. Home remedy users may hold negative views toward vaccines, such that a combination of little trust in the vaccine process and overestimation of the risk associated with the vaccine itself may contribute to vaccine hesitancy [7]. The belief that the use of complementary and alternative medicine (CAM) practices can eliminate the need for vaccination is related to reluctance to receive the recommended vaccination because of concerns and doubts about vaccines [8]. Parents who have not vaccinated their children appear to trust non-mainstream sources of information such as CAM practitioners [9].

In 2014 in South Tyrol, awareness and use of home remedies were investigated in a population-based cross-sectional telephone survey and results were presented at the annual conference of the German Society of General and Family Medicine (DGAM) in 2017; however, the study design and findings have not been fully published [10]. Since language group membership in South Tyrol was associated with home remedies’ awareness and use in the pre-pandemic study, these findings may contribute to a better understanding of the potential relevance of self-care in CAM and home remedies for local pandemic preparedness and experience.

Therefore, the main objective of this study was to assess and describe health self-management regarding the use of home remedies in the general population of South Tyrol. The aims were (1) to predict awareness of home remedies using socio-demographic characteristics (i.e., age, gender, educational level, region of origin, and language group), (2) to test the potential moderation effects of demographic characteristics and language group membership, (3) to explore patterns (i.e., latent classes) of home remedy use, and (4) to test the associations between socio-demographic characteristics and latent class membership.

## Methods

### Sample and Procedure

Data were derived from a population-based cross-sectional telephone survey study of health information-seeking behavior and awareness and use of home remedies initiated before the coronavirus pandemic because of the historically low vaccination rates in South Tyrol. Details of the study design have been previously reported [11]. This article focuses on the awareness and patterns of the use of home remedies. As described [11], the eligibility criteria for participants were living in South Tyrol, possessing a landline (only private households, no business phones), being at least 18 years old, and being declared to either the German or the Italian language group. According to data from the National Statistics Institute (www.dati.istat.it) for 2015, from a total of 210,000 private households in South Tyrol, 122,000 (58%) had a landline, 208,000 (99%) of private households had at least one mobile phone, and 92,000 (44%) used mobile phones. In the present study, only landline users were included, because mobile phone users cannot be limited to South Tyrol. Computer-assisted telephone interviews were conducted between August and September 2014 [11].

Data collection has also been described previously by Ausserhofer and co-workers [11]. The telephone survey was performed by Apollis (www.apollis.it), a private research institution in Bolzano (BZ), Italy, conducting empirical studies for public and private clients with a focus on education, labor market topics, active aging, and survey research. The goal was to conduct at least 500 interviews. Professional interviewers contacted a random sample of 1,445 households in South Tyrol with landline numbers. A total of 162 phone numbers were incorrect and 318 were not reachable. The remaining 965 were invited to participate in the telephone survey and appointments were made to answer the phone survey: 458 did not participate in the second call despite the appointment, for 46 no suitable appointment was found during the study period, 53 were not capable of participating, and 359 declined. Of the 507 phone interviews, three were excluded from the analyses as participants were not eligible, resulting in a total of 504 interviews. The interviews lasted from three to 28 min (mean,12 min) [11].

### Measurements

Awareness and use of home remedies were assessed using self-developed, single items. First, the participants were asked if they were using any home remedies. If they answered with yes, they were asked to mention the most commonly used home remedies In order to quantify the mentions, they were coded according to active ingredients, processing forms and application methods. For statistical analyses, nominations of herbal medicine and dietary supplements, and self-help practices [12, 13] were categorized in a list of 9 home remedies (0 = “no” and 1 = “yes”): (1) tea, (2) other plants, (3) compress, (4) crème, salve, (5) arnica, (6) alcohol, herb liqueur, (7) chamomile, (8) vinegar, oil, and (9) marigold. Participants’ sociodemographic factors included age (birth year), biological sex (male/female), mother’s language (German/Italian), educational level (highest degree), and region of origin (rural/urban).

### Statistical analysis

We used descriptive statistics, including means, standard deviations, frequencies, cross-tabulations, to describe the use of home remedies, and the characteristics of the sample. To avoid biases, sampling weights based on the age and sex distributions for the population of South Tyrol, according to the Provincial Statistics Institute for 2013, were employed for all analyses [2].

According to our research aims, we analyzed the data in four steps. First, we used logistic regression modeling to predict the prevalence of awareness of home remedies (0 = not aware of home remedies, 1 = aware of home remedies) based on the participants’ age (in years), biological sex (0 = male, 1 = female), educational level (0 = less than high school, 1 = high school +), region (0 = urban, 1 = rural), and language group (0 = Italian, 1 = German). The DFBETA statistics were used to evaluate the presence of potentially influential observations. Here, a respondent was flagged as a potentially influential observation if the DFBETA statistic exceeded threshold 2/√n [14]. The area under the receiver operating characteristic (ROC) curve (AUC) was used to evaluate the predictive accuracy of the multiple logistic regression model. Model fit was evaluated using the preudo-R2 coefficient of determination.

Second, follow-up moderation analyses were used to test the hypothesis that sociodemographic characteristics moderate the relationship between language group membership and awareness of home remedies. The Johnson-Neyman approach [15] was applied to post-hoc probe the moderation effects. Sampling weights based on age and sex distributions for the population were incorporated into all logistic regression models.

Third, we completed a latent class analysis (LCA) to explore whether meaningful latent classes of respondents’ home remedy use could be identified from the nine home remedies. LCA is a statistical model used to identify the underlying mutually exclusive and exhaustive subgroups of individuals with shared characteristics [16]. Because the number of latent classes was unknown a priori, a series of LC models with one to four latent classes were estimated. To avoid local maxima of log-likelihoods during model estimation, 1000 random starts were used for each model. To select the appropriate number of classes, Akaike information criterion (AIC) and Bayes information criterion (BIC) were applied (lower values indicate a better model fit). In addition, unadjusted and adjusted Lo-Mendell-Rubin (LMR) tests were used to evaluate whether the k-class solution was superior to the k – 1 class solution. A significant LMR test suggests that the k-class solution fits the data better than the k – 1 class solution. In addition to the statistical indices, the interpretability of the model coefficients was inspected for each model [17]. LCA assumes that the underlying latent classes explain the dependence structure of home remedy indicators (known as the local independence assumption). Standardized bivariate residuals were used to assess potential violations of the local independence assumption. Because language group-specific sampling weights were not available, LC modelling was performed using the total sample.

Fourth, after selecting the appropriate number of latent classes, a latent-class multinomial logistic regression model was used to predict latent-class memberships. Because a two-step approach (i.e., modally assigned LC memberships regressed on predictors in a multinomial logistic regression) is prone to overestimating the influence of predictors [18], a three-step approach was applied [19]. Here, a latent class model is first estimated, and in the second step, modally assigned LC memberships are extracted for each observation. In the third step, the measurement error in the modal assignments is considered by incorporating classification uncertainty rates in latent-class logistic regressions [19]. Respondents’ age (in years), biological sex (0 = male, 1 = female), education (0 = less than high school, 1 = high school +), region (0 = urban, 1 = rural), and language group (0 = Italian, 1 = German) were used to predict latent-class memberships. The level of significance was set at p < 0.05. Data analysis was conducted using Mplus version 7.3 [20]. When predicting latent class memberships, the main effects as well as potential moderation effects of covariates on the relationship between language groups and latent class memberships were investigated [21].

## Results

Characteristics of the study participants and representativeness of the sample are described in the first publication of the survey results on health information-seeking behavior [11]. Out of the 504 participants, 23 respondents (4.6%) had missing values in their demographic characteristics and were thus discarded from the analysis. Compared to participants with complete data, the subgroup with incomplete data was more likely to live in rural regions of South Tyrol (4 urban and 19 rural respondents; p = 0.035). For the analysis sample (n = 481), respondents’ (population-weighted) sociodemographic characteristics are summarized in Table 1.

**Table 1:** Descriptive statistics of the population weighted analysis sample (n = 481) by language group of the population-based cross-sectional telephone survey, August to September 2014, on awareness and use of home remedies in Italy’s alps

Variable	Language Group		
	German (*n* = 340)	Italian (*n* = 141)	Total (*n* = 481)
Age (years) *M* (*SD*)	47.8 (18.7)	51.1 (18.6)	48.8 (18.7)
Sex			
Male *n* (%)	167 (49.0)	70 (49.7)	237 (49.2)
Female	173 (51.0)	71 (50.3)	244 (50.8)
Educational level *n* (%)			
Less than high school	208 (61.2)	61 (43.5)	269 (56.0)
High school +	132 (38.8)	80 (56.5)	212 (44.0)
Region *n* (%)			
Urban	80 (23.3)	115 (81.7)	195 (40.4)
Rural	261 (76.7)	26 (18.3)	286 (59.6)
Home remedy awareness *n* (%)			
No	72 (21.0)	59 (41.7)	131 (27.1)
Yes	268 (79.0)	82 (58.3)	350 (72.9)

### Prediction of home remedies awareness

Out of the 481 participants, 357 (74.2%) reported to be aware of home remedies including herbal medicine, dietary supplements, and self-help practice. The mentions in German and Italian, and the relative nomination frequencies are shown in a Wordcloud graphic as Figure S1 and as Table S1 as supplementary material, respectively. Table 2 presents the logistic regression results. Overall, respondents from rural regions were less likely to use home remedies (OR = 0.35, 95% confidence interval [CI] = 0.19–0.67). In contrast, female (OR = 2.62, 95% CI = 1.69–4.10) as well as German-speaking participants (OR = 5.52, 95% CI = 2.91–9.88) were significantly more likely to use home remedies. No significant main effects were observed for participants’ age or educational level. The logistic regression model attained an area under the curve of AUC = 0.69 and a preudo-R2 of 0.10. Overall, two observations showed a DFBETA statistic greater than 2/√481 = 0.091. Temporarily discarding the two potentially influential observations had no substantive effects on the logistic regression results, and were therefore retained in the analysis sample.

**Table 2:** Results of the population weighted multiple logistic regression model to predict home remedies awareness (n = 481) in the population-based cross-sectional telephone survey, August to September 2014, in Italy’s alps

		95% Confidence Interval		
**Variables**	OddsRatio	lower	upper	*p*-value
Language group: German	5.25	2.91	9.88	< 0.001
Gender: Female	2.62	1.69	4.10	< 0.001
Age (in years)	1.00	0.99	1.01	0.717
Educational level: High school +	0.91	0.57	1.47	0.709
Region: Rural	0.35	0.19	0.64	0.001

**Fig. 1:**
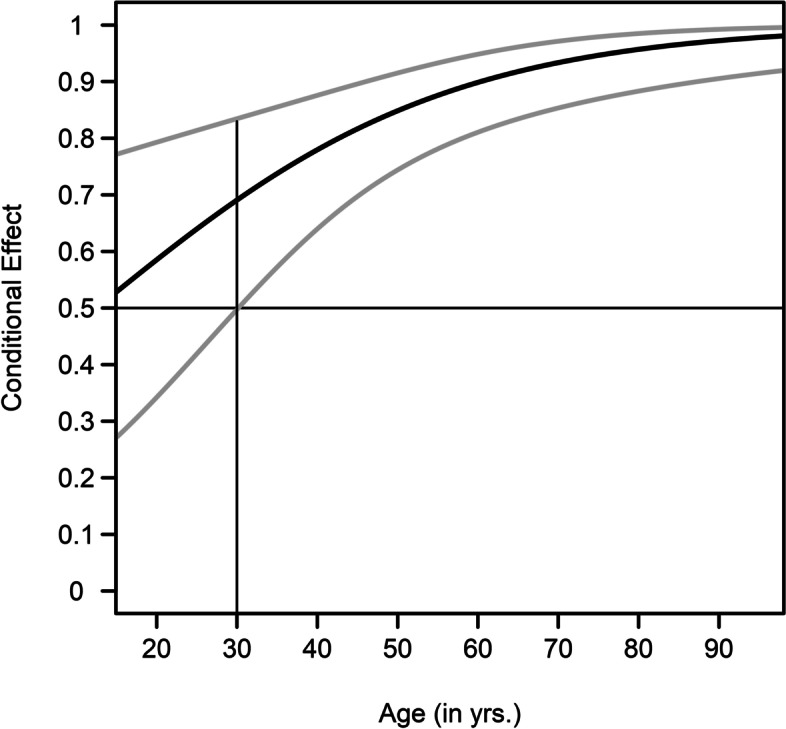
Moderating effect of age on the relation between language groups and home remedies use. At 30.1 + years of age (cf. vertical line), the association between being of German ethnicity and home remedies use is statistically significant. Gray lines give the 95% CI of the conditional effect (*n* = 481)

Follow-up moderation analysis suggested a significant interaction effect of age and language group (OR = 1.05, 95% CI = 1.02–1.08). No significant interaction effects were observed for biological sex, education, or region (all p > 0.21). After including the “age × language group” interaction term, the area under the curve increased to 0.71 and pseudo-R2 increased to 0.12. Figure 1 shows the conditional effect of being of German ethnicity on the probability of using home remedies as a function of the participants’ age. the Johnson-Neyman technique suggested a significant increase in the probability of using home remedies for participants older than 30.1 years of age. In contrast, the conditional effects were insignificant for participants ≤ 30.1 years of age.

### Patterns of home remedies use

Among the 357 participants that reported use of home remedies, the three most frequent remedies were “teas” (48.2%), “plants” (21.0%), and “compresses” (19.5%). The three least frequently used home remedies were “chamomile” (13.3%), “vinegar, oil” (13.2%), and “marigold” (6.8%; cf. Table 3). Four observations had missing values for home remedy indicators. Full-information maximum likelihood estimation was applied to handle these missing data points.

**Table 3:** Descriptive statistics of population weighted sample that reports home remedy use by language group of the population-based cross-sectional telephone survey, August to September 2014, in Italy’s alps

	Language Group		
**Variable**	German (*n* = 273)	Italian (*n* = 84)	Total (*n* = 357)
Age (years) *M* (*SD*)	48.9 (17.2)	47.6 (19.2)	48.6 (17.7)
Sex *n* (%)			
Male	124 (45.2)	30 (35.8)	154 (43.0)
Female	150 (54.8)	54 (64.2)	204 (57.0)
Educational Level *n* (%)			
Less than high school	166 (60.8)	37 (43.8)	203 (56.8)
High school +	107 (39.2)	47 (56.1)	154 (43.2)
Region *n* (%)			
Urban	72 (26.3)	75 (89.2)	146 (41.0)
Rural	202 (73.7)	9 (10.8)	211 (59.0)
Home Remedy Use *n* (%)			
Tea	148 (41.5)	24 (6.7)	172 (48.2)
Plants	64 (17.9)	11 (3.2)	75 (21.0)
Compresses	59 (16.5)	11 (3.0)	70 (19.5)
Crème, Salve	61 (17.0)	4 (1.2)	65 (18.2)
Arnica	52 (14.5)	4 (1.1)	56 (15.6)
Alcohol, Herb Liqueur	45 (12.5)	8 (2.3)	53 (14.8)
Chamomile	41 (11.5)	6 (1.8)	48 (13.3)
Vinegar, Oil	42 (11.7)	5 (1.5)	47 (13.2)
Marigold	24 (6.8)	0 (0)	24 (6.8)

*n* = frequencies, *M* = mean, *SD* = standard deviation

The LC model fit indices and estimated class sizes (based on modal assignment) for k = 1–4 latent classes are summarized in Table 4. Sampling weights were incorporated into all LC models. In general, the AIC values decrease with every additional class, which hampers distinct model selection. Because this is also in line with the observation that AIC may tend to overestimate the number of latent classes, we primarily focused on BIC. BIC favored the 2-class solution. The adjusted and unadjusted LMR tests also suggested that the 2-class solution was sufficient to explain the dependence structure of home remedy indicators, which was confirmed by an excellent model fit of the 2-class solution (Pearson χ2(492) = 440.6, p = 0.953). Thus, we decided to retain the population weighted 2-class solution as the final model. The bivariate standardized residuals of the selected 2-class model were inspected to evaluate the local dependence assumption. Based on Bonferroni-adjusted tests (i.e., based on 36 (number of item pairs) × 4 (number of comparisons per item pair) = 144 tests, an item pair was considered conspicuous when the standardized residuals exceed a z-score of 3.58), only the item pair “marigold – crème, salve” (z = 5.29) was identified as potentially being conspicuous. Re-estimating the weighted 2-class model while allowing this residual covariance, did not significantly change the results. Thus, the initial 2-class model parameters are reported.

**Table 4:** Summary of latent class model fit for weighted sample of the population-based cross-sectional telephone survey, August to September 2014, on awareness and use of home remedies in Italy’s alps (n = 357, AIC = Akaike Information Criterion, BIC = Bayes Information Criterion, LMR = p-value of the Lo-Mendel-Rubin test, adj. LMR = p-value of the adjusted LMR; indices suggesting best model fit are marked bold)

					Latent class sizes based on modal assignment			
No. of classes	AIC	BIC	LMR	adj. LMR	LC1	LC2	LC3	LC4
1	2916	2950	-	-	357	-	-	-
2	2830	**2904**	**0.005**	**0.006**	77	280	-	-
3	2804	2916	0.2653	0.2698	54	250	53	-
4	**2794**	2945	0.7127	0.7159	59	58	234	6

**Fig. 2:**
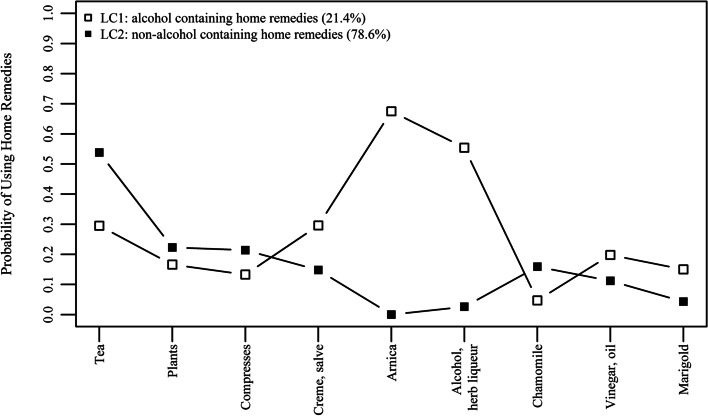
Patterns of home remedies use for two latent classes. Latent Class 1 (LC1): “alcohol containing home remedies” pattern; Latent class 2 (LC2): “non-alcohol containing home remedies”. (*n* = 357)

LC-specific patterns of home remedy use are summarized in Fig. 2 and can be described as either “alcoholic-containing home remedies” (n = 77; 21.4%) and “non-alcohol-containing home remedies (n = 280; 78.6%). Members of the “alcoholic-containing home remedies” most likely used arnica and alcohol/herb liqueur. The “non-alcohol-containing home remedies” group was more likely to use home remedies, such as teas, plants, compresses, and chamomile, but reported to almost never use arnica and alcohol/herb liqueur.

In the next step, covariates were entered into the LC model to predict latent class membership using the 3-step latent logistic regression approach described above. The descriptive characteristics of the two latent classes are summarized in Table 5. In addition to testing the main effects on class membership, all potential two-way interactions were investigated [21]. Non-significant predictors were omitted from the model because of parsimony. No significant differences were observed in terms of age or educational level. In addition, none of the potential two-way interactions was significant. Compared to the “non-alcohol containing home remedies” class, members from the “alcoholic-containing home remedies” class were more likely to be male (OR = 1.98, 95% CI = 1.01–3.89), more likely to be of German ethnicity (OR = 4.31, 95% CI = 1.30–14.23), and more likely to live in an urban region (OR = 2.50, 95% CI = 1.07–5.85).

**Table 5:** Descriptive statistics of weighted sample by latent class membership of the population-based cross-sectional telephone survey, August to September 2014, on awareness and use of home remedies in Italy’s alps (n = 357)

Variable		Latent Classes	
		LC1: “Alcohol-containing home remedies”	LC2: “Non-alcoholic home remedies”
Class Size	*n* (%)	77 (21.4)	280 (78.6)
Sex	*n* (%)		
Male		42 (55.1)	111 (39.7)
Female		34 (44.9)	169 (60.3)
Ethnicity	*n* (%)		
German		66 (86.2)	207 (73.9)
Italian		11 (13.8)	73 (26.1)
Educational Level	*n* (%)		
less than high school		43 (56.0)	160 (56.1)
high school +		34 (43.9)	121 (43.0)
Region	*n* (%)		
urban		35 (45.8)	111 (39.7)
rural		41 (54.2)	169 (60.3)
Age (years)	*M* (*SD*)	50.6 (16.7)	48.0 (17.9)

*n* = frequencies, *M* = mean, *SD* = standard deviation

## Discussion

The study showed that more than two-thirds of the population in South Tyrol (southern alps, Italy) reported using home remedies. Multiple logistic regression suggested that participants from rural regions were less likely, and female and German-speaking participants were more likely to use home remedies. In addition, follow-up moderation analysis suggested a significant interaction effect of participants’ age and being of German ethnicity. Here, the conditional effect of language group membership on home remedy use reached significance for participants older than 30.1 years of age.

In European high-income countries, awareness and use of home remedies have reached frequencies up to 75% of the populations. A study carried out in 2014 in Germany showed that approximately 80% of patients in general practice used home remedies [22]. In 2020 in Switzerland, home remedies were used by 65% of patients in general practice [23]. Several studies have shown that home remedies are used more frequently by female patients [22, 24, 25]. Common characteristics of CAM users include female gender, employment, higher education, private health insurance coverage, and higher-than-average incomes [26]. Female gender and higher socioeconomic status predict experience with CAM without predicting approval of CAM [27]. Women living in urban areas, highly educated, aged more than 40 years, who suffer from severe chronic back pain, were more inclined to go to CAM therapists [28], although female acupuncture users with osteoarthritis were more likely to reside in non-urban areas than those who did not use acupuncture [29]. In our study, awareness and use of home remedies were higher in urban than in rural regions and in women. In Italy, similar characteristics have been previously observed for CAM use in cancer patients in Tuscany [30].

In South Tyrol, due to the region’s high plant diversity and relatively isolated population, unique traditional botanical knowledge of medicinal plants has flourished, which traces its history back to prehistoric times [31]. Not surprisingly, among the 357 participants who reported use of home remedies in our study, the two most common reported home remedies were teas (48.2%) and plants (21.0%).

Two latent classes of home remedies were observed: “alcoholic home remedies” (21.4%; predominantly using arnica and alcohol/herb liqueur) and “non-alcohol-containing home remedies” (78.6%; almost never using arnica and alcohol/herb liqueur). Compared to the “non-alcohol-containing home remedies” class, members of the “alcoholic home remedies” class were more likely to be male, live in an urban region, and be German speakers. A European survey revealed that alcohol—either drunk alone or added to other liquids—is used as home remedy by 12% of respondents [32]. In a study on self-care for common colds by primary care patients, women reported a greater variety of self-care items than men; however, more men reported using alcohol (17.8% vs. 8.4%, p < 0.001) [33].

Although surveys carried out in Western countries tend to show that a large part of the population regularly uses home remedies and that patients would like to be better informed by their general practitioners (GPs) about the use of these remedies [22, 34, 35], little is known about the prevalence and patterns across Europe. A cross-national study on self-care for the common cold, including home remedies, showed a similar pattern but quantitative differences across sites in 14 European countries (including Italy and Austria) [32]. Our finding that alcoholic home remedies are associated with language group membership suggests that awareness and use of home remedies are culturally sensitive even at the regional level within high-income countries.

Vaccine hesitance and CAM, including home remedies, have been linked both, at the population level, as well as among healthcare providers. For example, for influenza-like syndromes in Italy, only 14% of the general population received influenza vaccination annually, and almost 60% had never received vaccination, whereas approximately 36% of respondents regarded homeopathy as a helpful alternative because of perceived as safer [36]. Among French GPs, who are the first care option in infectious diseases, vaccine hesitancy was identified in up to 15% of cases and was associated with higher frequencies of occasional practice of CAM as compared to GPs who were not or only slightly vaccine-hesitant [37]. Adult population vaccination by GPs in Germany was linked to hesitant GPs’ self-vaccination behavior, which was independently associated with being a homeopathic GP [38].

Prior to the coronavirus pandemic, surveys have investigated community experience for pandemic preparedness and identified home remedies as a widely reported treatment [39]. A comprehensive study to evaluate biologically based CAM therapies such as herbs, foods, and supplements during the coronavirus pandemic via analysis of Google search engine statistics revealed that Google users in the USA and Great Britain displayed more potential interest than those in France, Germany, and Italy [40]. Awareness and use of home remedies is frequent in South Tyrol, and prevalence rates have independently increased for women, urban inhabitants, and German speakers, according to this survey. As the coronavirus vaccination rate distribution patterns between German- and Italian-speaking inhabitants in December 2021 coincided with this distribution characteristics of awareness and use of home remedies, it cannot be excluded that vaccination hesitance and CAM are also linked in South Tyrol, however, further studies are necessary to directly test this hypothesis.

A characteristic of the South Tyrolean society is its relatively strong ethnicization. This is primarily reflected in the institutionalized separation by language groups (e.g. linguistically separate school systems, allocation of public service jobs according to “ethnic proportionality”), but can also be clearly demonstrated in the economic sphere (agriculture and tourism as traditionally “German” domains, public service and industry for a long time as the primary economic base of the “Italians”). This ethnicization at its core is based on the economic differences of interest between the language milieus [41, 42]. It is reasonable to assume that this language group differences also affect home remedy and CAM use.

CAM use was an important predictor of changing to a healthier lifestyle during the first wave of the coronavirus pandemic and was not statistically significant associated with a change to an unhealthy lifestyle [43]. Since not using CAM was positively associated with GPs consultations [44], awareness and use of home remedies may help avoid unnecessary medical consultations. However, it may also indicate trust in and adherence to conventional medicine and support for preventive health measures, including vaccination. Although CAM literature cannot be simply described as being either pro- or antivaccination without considering finer areas of disagreement [45, 46], CAM has emerged as part of an expert system countering conventional medicine, and pro-vaccination health professionals, policymakers, and information providers seeking to address the role of CAM in vaccine rejection face significant challenges due to the epistemic basis of its proponents’ decisions [47]. Engaging with CAM providers appears to be a feasible and innovative avenue for providing vaccine information and designing communication tools aimed at vaccine-hesitant healthcare providers [48].

This study has several limitations. (i) The study database was representative of the adult population living in private households and using landline phones in South Tyrol in 2014. Smartphone ownership may have been underrepresented in this study because of design-related selection bias that may have impacted the awareness and use of home remedies [49]. The profile of the population that has only a mobile phone differs from that with a landline telephone. A study performed in Spain showed that persons contacted through a mobile phone were more likely to be a foreigner, to belong to the manual social class, to have a lower educational level, and to be a smoker than those contacted through a landline telephone [50]. (ii) Awareness and use of home remedies for children and adolescents (> 18 years old) were not assessed because of the inclusion criteria. (iii) A self-reporting design was used where the respondents identified what home remedies they used. This makes it impossible to directly compare the data with results of home remedy studies in which standardized measurement methods such as the international questionnaire to measure usage of complementary and alternative medicine (I-CAM-Q) [12, 13] was used. (iv) In the statistical analysis, the awareness and use of home remedies and additional parameters were dichotomized. Response categories were collapsed into two categories because latent class modeling based on the original ordinal scales would require larger sample sizes to guarantee model convergence. (v) The survey was performed in 2014; characteristics of use and awareness of home remedies may have changed until today. Furthermore, differences in knowledge and use of home remedies between members of different language groups are not surprising. However, the possibility that this socio-cultural disparity could translate into quite different vaccination behaviors in an economically well-developed, central European region with a relatively small catchment area gave a whole new meaning to the relatively old 2014 data. We therefore decided to conduct the analyses presented and to publish the results.

## Conclusion

Disease prevention and self-management with the use of CAM is not negative per se as long as important preventive and diagnostic measures are not neglected, which seems to be frequently the case during the coronavirus pandemic. Language group membership determines the awareness and use of home remedies in Italian alps. In South Tyrol, compared to Italian speakers, German speakers’ greater awareness and more frequent use of home remedies may theoretically be linked to increased vaccine hesitancy and low vaccination rates of mandatory vaccination of children as well as vaccination against COVID-19 because of corresponding language distribution patterns. If the observed associations with home remedies, however, explain the lower vaccination rates in South Tyrol among German speakers requires further study.

## Supplementary Information

Supplementary Material 1

## Data Availability

The datasets used and/or analyzed during the current study are available from the corresponding author upon reasonable request. The datasets generated and/or analyzed during the current study are not publicly available because the survey results could be misused for market research purposes.
