# Supplementary material for: Awareness and use of home remedies in Italy’s alps: a population-based cross-sectional telephone survey
Source: BMC Complement Med Ther. 2022 Nov 11;22:292. doi: 10.1186/s12906-022-03781-0 (PMC9650663; doi:10.1186/s12906-022-03781-0)
Supplement: Supplementary file 1 — Supplementary Material 1 [file 12906_2022_3781_MOESM1_ESM.docx]

Supplementary Material

*Research Article*

Awareness and use of home remedies in Italy’s alps: A population-based cross-sectional telephone survey

Wolfgang Wiedermann et al.

**Figure S1.** Wordcloud of all named home remedies in a population-based cross-sectional telephone survey of South Tyrol, Italy, August to September 2014. The font size corresponds to the frequency with which the words are mentioned. The proximity of the words to each other is random. The word list in German and Italian was cleaned for irrelevant filler words, declensions and different spellings.

**Table S1.** Mentions as home remedies of herbal medicine, dietary supplements and self-help practices in a population-based cross-sectional telephone survey of South Tyrol, Italy, August to September 2014.

| **Home Remedies** | **Mentions***  **N (%)** |
| --- | --- |
| **Herbal Medicine and Dietary Supplements** |  |
| Teas | 171 (48) |
| Arnica | 57 (16) |
| Herbs general | 54 (15) |
| Liquor, Alcohol | 54 (15) |
| Chamomile | 46 (13) |
| Vinegar, Oils | 46 (13) |
| Onion | 39 (11) |
| Honey | 39 (11) |
| Sage | 29 (8) |
| Marigold | 25 (7) |
| St. John's wort | 21 (6) |
| Salts, Minerals | 21 (6) |
| Milk | 11 (3) |
| Other plants | 75 (21) |
| Other | 31 (9) |
| **Self-help Practice** |  |
| Compresses, rubbing | 68 (19) |
| Cream, Ointment | 64 (18) |
| Inhalation | 36 (10) |
| Homeopathy | 18 (5) |
| Aspirin or similar drug | 7 (2) |
| Other | 12 (3) |

*Mentions by percent of study participants were coded by active ingredients, processing forms and application methods, e.g., peppermint tea, the vapors of which are inhaled, would be coded as "peppermint" as the active ingredient, "tea" as the form of preparation and "inhalation" as the form of administration, if accordingly indicated.
